# Plasma Membrane Na^+^-Coupled Citrate Transporter (SLC13A5) and Neonatal Epileptic Encephalopathy

**DOI:** 10.3390/molecules22030378

**Published:** 2017-02-28

**Authors:** Yangzom D. Bhutia, Jonathan J. Kopel, John J. Lawrence, Volker Neugebauer, Vadivel Ganapathy

**Affiliations:** 1 Department of Cell Biology and Biochemistry, Texas Tech University Health Sciences Center, Lubbock, TX 79430, USA; yangzom.d.bhutia@ttuhsc.edu; 2 Department of Pharmacology and Neuroscience, Texas Tech University Health Sciences Center, Lubbock, TX 79430, USA; jonathan.kopel@ttuhsc.edu (J.J.K.); john.lawrence@ttuhsc.edu (J.J.L.); volker.neugebauer@ttuhsc.edu (V.N.); 3 Center of Excellence for Translational Neuroscience and Therapeutics, Texas Tech University Health Sciences Center, Lubbock, TX 79430, USA

**Keywords:** citrate transporter, NaCT (SLC13A5), CIC (SLC25A1), cytoplasmic citrate, mitochondrial citrate, GABA, neurotransmitters, fatty acid synthesis, cholesterol synthesis, tricarboxylic acid cycle, NMDA receptor, zinc

## Abstract

SLC13A5 is a Na^+^-coupled transporter for citrate that is expressed in the plasma membrane of specific cell types in the liver, testis, and brain. It is an electrogenic transporter with a Na^+^:citrate^3−^ stoichiometry of 4:1. In humans, the Michaelis constant for SLC13A5 to transport citrate is ~600 μM, which is physiologically relevant given that the normal concentration of citrate in plasma is in the range of 150–200 μM. Li^+^ stimulates the transport function of human SLC13A5 at concentrations that are in the therapeutic range in patients on lithium therapy. Human SLC13A5 differs from rodent Slc13a5 in two important aspects: the affinity of the human transporter for citrate is ~30-fold less than that of the rodent transporter, thus making human SLC13A5 a low-affinity/high-capacity transporter and the rodent Slc13a5 a high-affinity/low-capacity transporter. In the liver, SLC13A5 is expressed exclusively in the sinusoidal membrane of the hepatocytes, where it plays a role in the uptake of circulating citrate from the sinusoidal blood for metabolic use. In the testis, the transporter is expressed only in spermatozoa, which is also only in the mid piece where mitochondria are located; the likely function of the transporter in spermatozoa is to mediate the uptake of citrate present at high levels in the seminal fluid for subsequent metabolism in the sperm mitochondria to generate biological energy, thereby supporting sperm motility. In the brain, the transporter is expressed mostly in neurons. As astrocytes secrete citrate into extracellular medium, the potential function of SLC13A5 in neurons is to mediate the uptake of circulating citrate and astrocyte-released citrate for subsequent metabolism. *Slc13a5*-knockout mice have been generated; these mice do not have any overt phenotype but are resistant to experimentally induced metabolic syndrome. Recently however, loss-of-function mutations in human SLC13A5 have been found to cause severe epilepsy and encephalopathy early in life. Interestingly, there is no evidence of epilepsy or encephalopathy in *Slc13a5*-knockout mice, underlining the significant differences in clinical consequences of the loss of function of this transporter between humans and mice. The markedly different biochemical features of human SLC13A5 and mouse Slc13a5 likely contribute to these differences between humans and mice with regard to the metabolic consequences of the transporter deficiency. The exact molecular mechanisms by which the functional deficiency of the citrate transporter causes epilepsy and impairs neuronal development and function remain to be elucidated, but available literature implicate both dysfunction of GABA (γ-aminobutyrate) signaling and hyperfunction of NMDA (*N*-methyl-d-aspartate) receptor signaling. Plausible synaptic mechanisms linking loss-of-function mutations in SLC13A5 to epilepsy are discussed.

## 1. Introduction

**Figure 1:**
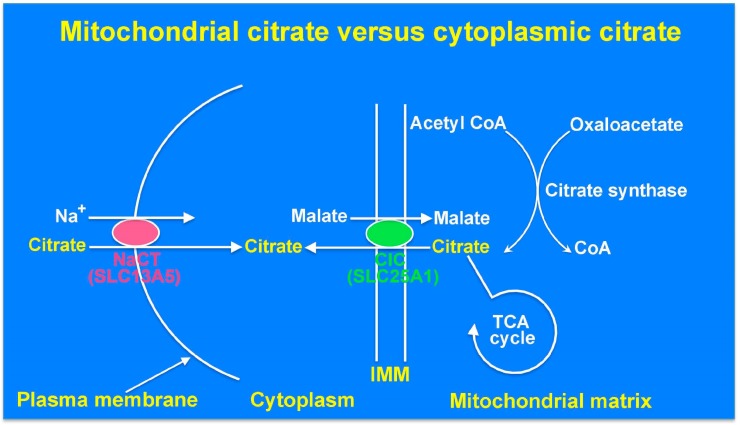
Roles of the mitochondrial citrate transporter SLC25A1 and the plasma membrane citrate transporter SLC13A5 as the determinants of citrate levels in the mitochondrial matrix and the cytoplasm. NaCT, Na^+^-coupled citrate transporter; CIC, citrate carrier; CoA, coenzyme A; TCA, tricarboxylic acid cycle; IMM, inner mitochondrial membrane.

Citrate is a key metabolite, which is at the junction of many important metabolic pathways. The most widely known function of citrate is its role in the tricarboxylic acid (TCA) cycle where it serves as the starting point for the generation of reducing equivalents NADH and FADH_2_, which then enter the electron transport chain to generate ATP (Figure 1). This citrate functioning in the TCA cycle is generated within the mitochondrial matrix via citrate synthase, which uses acetyl CoA and oxaloacetate as substrates to synthesize citrate. In the well-fed state with sufficient cellular ATP levels, cells switch from a catabolic phenotype to an anabolic phenotype, thus suppressing the TCA cycle and using citrate for anabolic reactions instead. Even though citrate has multiple biological functions, all of these functions except for its role in the TCA cycle occur in the cytoplasm. This necessitates the transfer of citrate from the mitochondrial matrix into the cytoplasm. This transfer is mediated by a specific transporter present in the inner mitochondrial membrane (SLC25A1), which mediates the efflux of citrate from the matrix into the cytoplasm in exchange for malate from the cytoplasm into the matrix. Once delivered into the cytoplasm, citrate inhibits catabolism of glucose by inhibiting the key rate-limiting enzyme phosphofructokinase-1 in glycolysis. Citrate in the cytoplasm also serves as the carbon source for the synthesis of fatty acids and cholesterol; this occurs via the cleavage of citrate into acetyl CoA and oxaloacetate by ATP:citrate lyase. Acetyl CoA is the starting molecule for the fatty acid synthase complex in the cytoplasm where the two carbons present in acetyl CoA are added sequentially to generate long-chain fatty acids with malonyl CoA as an intermediate generated from acetyl CoA by acetyl CoA carboxylase. Acetyl CoA is also the starting molecule for cholesterol biosynthesis via the generation of the key intermediate hydroxymethylglutaryl CoA (HMG-CoA) from three molecules of acetyl CoA by the enzymes thiolase and HMG-CoA synthase.

It is widely believed that the cytoplasmic citrate, which serves a key role in the regulation of glycolysis and in the synthesis of fatty acids and cholesterol, originates solely from the mitochondrial matrix. However, plasma contains significant amounts of citrate; under physiological conditions, the levels of citrate in the circulation are in the range of 150–200 μM [1,2], the second highest in terms of concentrations among the various monocarboxylates and TCA cycle intermediates (Table 1). Only lactate is present in plasma at concentrations higher than citrate. All of these metabolic intermediates are energy rich, meaning that they have the potential to generate significant amounts of metabolic energy in the form of ATP (Table 1). This raises the question as to whether the citrate present in the circulation plays any role as a potential source of cytoplasmic citrate. A definitive answer to this question would obviously depend on whether or not mammalian cells express a citrate transporter in the plasma membrane. The presence of such a transporter in mammals would make sense given the high levels of citrate in the circulation and also given the fact that plasma membrane transporters have been identified in mammalian cells for all other monocarboxylates and TCA cycle intermediates. Lactate and pyruvate are transported across the plasma membrane in mammalian cells by H^+^-coupled monocarboxylate transporters (MCTs belonging to the *SLC16* gene family) [3] and Na^+^-coupled monocarboxylate transporters (SMCTs belonging to the *SLC5* gene family) [4,5]. The dicarboxylate intermediates of the TCA cycle such as succinate and fumarate are transported across the plasma membrane by Na^+^-coupled dicarboxylate transporters (NaDCs belonging to the *SLC13* gene family) [6]. However, none of these transporters prefers citrate as a substrate, thus leaving the issue of whether or not mammalian cells express a transporter for citrate in the plasma membrane unresolved.

## 2. Identification and Molecular Characterization of the Plasma Membrane Citrate Transporter SLC13A5

The search for the first transporter for citrate in the plasma membrane of mammalian cells began with the identification of a transporter in *D. melanogaster* that showed a marked impact on the life span of the organism. In 2000, Rogina et al. [7] reported that certain Drosophila mutants had significantly extended life span and that heterozygous loss in the expression of a transporter gene was responsible for this phenomenon. The authors named the transporter Indy (I’m not dead yet). This original report did not identify the function of the transporter but found the transporter to be structurally similar to the two transporters in mammalian cells, namely NaDC1 (SLC13A2) and NaDC3 (SLC13A3), which mediate Na^+^-coupled uptake of various dicarboxylate intermediates of the TCA cycle across the plasma membrane. Based on this structural similarity, the authors postulated that the transporter in Drosophila whose partial loss of function leads to life span extension most likely mediates the cellular uptake of TCA cycle intermediates. It was thought that the mechanistic connection between the partial loss of function and life span extension was based on the transporter-dependent changes in mitochondrial function. Mitochondria are dynamic organelles that play an obligatory role in the generation of metabolic energy and hence in the survival of most cells. The TCA cycle intermediates fuel this mitochondrial metabolism. However, mitochondria also are the site at which a considerable amount of highly reactive oxygen radicals (superoxide, hydrogen peroxide, and hydroxyl radical) is generated as the unintended side products of the electron transport chain that generates ATP. As such, while the generation of ATP makes the mitochondria obligatory for survival and life, the generation of reactive oxygen species that damage cellular macromolecules such as DNA/RNA, proteins, and lipids makes the same organelles detrimental to the organism. In fact, continued function of mitochondria is believed to contribute to the cumulative accumulation of cellular damage, thus resulting in ageing and ultimately death. Therefore, suppression of mitochondrial function could decrease the cellular damage because the decreased generation of reactive oxygen radicals and thus potentially extend the life span. There is substantial experimental evidence in support of this idea. Caloric restriction, which suppresses mitochondrial function, is a well-documented approach to extend the life span in animals and humans [8,9]. If Drosophila Indy is indeed a transporter for TCA cycle intermediates in the plasma membrane, heterozygous loss of its expression would result in decreased availability of substrates for mitochondrial metabolism, thus creating a cellular environment akin to caloric restriction and providing a molecular basis of life span extension in heterozygous mutants [10,11].

At the time when the discovery of Drosophila Indy appeared in the literature, there were only two transporters in mammalian cells capable of mediating the uptake of TCA cycle intermediates across the plasma membrane; these were NaDC1 (SLC13A2) and NaDC3 (SLC13A3). This was the reason why Rogina et al. [7] focused on the sequence similarity of Drosophila Indy with that of SLC13A2 and SLC13A3. If Indy is a transporter for TCA cycle intermediates, which one of these two transporters represents the mammalian ortholog of Drosophila Indy? Even though NaDC1, as well as NaDC3, transport various dicarboxylate intermediates of the TCA cycle in a Na^+^-dependent manner, there are significant differences between the two transporters in kinetic features. Therefore, it was difficult to determine if Drosophila Indy is related to NaDC1 or NaDC3 without any information on the transport function of the Drosophila transporter. Subsequent studies in our lab and in others delineated the transport function of Drosophila Indy [12,13]. As predicted, Indy was indeed a transporter for TCA cycle intermediates; it transports several dicarboxylate intermediates of the TCA cycle. However, there were important and surprising differences between Drosophila Indy and mammalian NaDC1/NaDC3. The transport function of Drosophila Indy was Na^+^-independent in contrast to the Na^+^-dependent mammalian transporters NaDC1 and NaDC3. More importantly, Drosophila Indy accepted the tricarboxylate TCA cycle intermediate citrate with higher affinity than the dicarboxylate intermediates of the TCA cycle; this is certainly not the case with NaDC1 and NaDC3, which prefer dicarboxylates to tricarboxylates. This indicated that neither NaDC1 nor NaDC3 is likely to be the mammalian ortholog of Drosophila Indy.

Then, the question was: do mammalian cells express a plasma membrane transporter for citrate similar to Drosophila Indy? A quest to answer this question in our laboratory led to the discovery of the plasma membrane citrate transporter in mammals [14]. We identified a partial mRNA sequence in the EST (Established Sequence Tags) database that was expressed in the rat brain and coded for a partial protein sequence homologous to Drosophila Indy but different from the sequences of rat NaDC1 and rat NaDC3. Using the nucleotide sequence of this EST, we designed a cDNA probe to screen a rat brain cDNA library, which led to isolation of the full-length clone for the transporter. In heterologous expression systems, this transporter preferentially transported citrate [14]. The dicarboxylate intermediates of the TCA cycle were also substrates for this mammalian transporter, but their affinities were much lower than that for citrate. However, even though the substrate specificity of the rat citrate transporter was similar to that of Drosophila Indy, the mammalian transporter was obligatorily dependent on Na^+^ unlike the Na^+^-independent Drosophila Indy. Based on the features of the transport function, the newly identified rat transporter was named NaCT (Na^+^-coupled citrate transporter). According to the Human Genome Nomenclature, NaCT is identified as SLC13A5. Northern blot analysis indicated the presence of Slc13a5 transcripts in the liver, brain, and testis. Subsequently, we cloned this transporter from humans [15], mice [16], dogs, rabbits, monkeys, chimpanzees, and zebrafish [17]. NaCT (SLC13A5) represents the first Na^+^-coupled transporter selective for citrate that is expressed in the plasma membrane in mammalian tissues (Figure 1).

## 3. Cellular and Subcellular Localization of NaCT Protein

As the expression of the transporter is mostly restricted to three tissues, namely the brain, liver, and testis, we focused on the cellular and subcellular localization of the transporter protein in these tissues. In the rat brain, the protein is expressed exclusively in neurons cultured in vitro; astrocytes are negative for the protein [18]. Primary cultures of neurons from human brain cortex also express the transporter, demonstrable at the mRNA level and functional level (S. Ramachandran and V. Ganapathy, unpublished data). It has to be emphasized, however, that there are no published reports on the expression of the transporter in human brain tissue in situ. In the liver, the transporter is expressed in hepatocytes where it localizes specifically to the sinusoidal membrane that is in contact with blood [19]. In the testis, the transporter is expressed only in germ cells; in human spermatozoa, we found the expression only in the mid piece where mitochondria are located (P. M. Martin and V. Ganapathy, unpublished data).

## 4. Functional Differences between Human and Rodent NaCTs

Even though the citrate transporters cloned from all vertebrate species are obligatorily dependent on Na^+^ for their transport function and are electrogenic, we found important and physiologically relevant differences in kinetic features between the human and rodent transporters [14,15,16]. The Michaelis constant for the transporter to transport citrate is ~20 μM in mice and rats [14,16], whereas the corresponding value for the human and other primate transporters is at least 30-fold higher [15]. As the concentration of citrate in circulation is in the range of 150–200 μM, the rodent transporters operate under physiological conditions in a fully saturated manner; in contrast, these physiological concentrations of citrate in plasma represent sub-saturating levels for the human transporter. This is an important distinction in terms of the capacity of the human and rodent NaCTs to transport citrate from the circulation into cells. As the rodent transporters are saturated at low micromolar concentrations of citrate, they represent a low-capacity transport system; as the human transporter functions at sub-saturating level at physiological concentrations of citrate in blood, it represents a high-capacity transport system. This means that, at normal concentrations of citrate in the circulation, the amount of citrate transported into cells per transporter molecule is much lower in rodents than in humans.

In addition to this important difference in the kinetic features of the transporter between humans and rodents, we found another interesting distinction. The human transporter and the rodent transporters do not accept Li^+^ in place of Na^+^ as the co-transported ion [14,15,16]; however, in the presence of Na^+^, the rodent transporters are inhibited by Li^+^, whereas the human transporter is stimulated by Li^+^ [20]. The stimulatory effect of Li^+^ is conserved in two other primates, namely monkeys and chimpanzees [17]. The Li^+^ effect on human NaCT is pharmacologically and clinically relevant for two reasons. First, the stimulation occurs at Li^+^ concentrations found in patients on lithium therapy; the therapeutic range for plasma concentration of Li^+^ is ~2 mM, a concentration at which human NaCT is stimulated at least three-fold. Second, lithium therapy is associated with dyslipidemia and body weight gain [21,22], but the molecular mechanisms underlying these side effects remain largely unknown. The activation of the transport function of human NaCT in the liver might be relevant to this phenomenon. It has been documented in cell cultures that NaCT expressed in the sinusoidal membrane of the hepatocytes transport citrate from the extracellular medium for subsequent use in fatty acid biosynthesis; a similar process might occur in vivo where the transporter facilitates active transfer of circulating citrate into hepatocytes for fat (fatty acids, triglycerides, and cholesterol) biosynthesis. This pathway is likely to be activated markedly in patients on lithium therapy, thus resulting in increased use of extracellular citrate for hepatic biosynthesis of fat and hence in increased production of low-density lipoproteins. This is, however, only speculative at present. Unfortunately, this issue cannot be addressed using rodents as animal models because NaCTs in these species are affected by Li^+^ in a manner that is quite opposite of human NaCT.

## 5. Transcriptional Regulation of NaCT Expression

The stimulatory effect of Li^+^ on NaCT occurs at the protein level; the catalytic activity of the transporter is increased in the presence of Li^+^. Recent studies have uncovered regulatory mechanisms that are involved in the transcriptional activation of the gene coding for the transporter. SLC13A5 is induced in human liver cells by pregnane X receptor (PXR) [23] and in rat liver cells by aryl hydrocarbon receptor (AhR) [24]. The promoter of SLC13A5 contains binding sites for both of these nuclear receptors. Both PXR and AhR are well known for their impact on lipid and energy metabolism; ligand-dependent activation of these two receptors promote lipid synthesis in the liver and consequently hepatic steatosis. As NaCT facilitates the utilization of circulating citrate for lipid synthesis in the liver, induction of this transporter in hepatocytes by PXR and AhR ligands at least partly contributes to this phenomenon. Indeed, activation of these receptors in hepatocytes with respective ligands promotes the utilization of extracellular citrate for lipid synthesis. SLC13A5 is also subject to regulation by glucagon in rat liver cells [25]; this involves intracellular signaling via cAMP. As the glucagon/insulin ratio in terms of biological activity is increased in uncontrolled type 2 diabetes, the glucagon-dependent induction of SLC13A5 expression in liver cells might contribute to hyperlipidemia associated with diabetes. Recent studies have shown that expression levels of this transporter in human liver might be associated with non-alcoholic fatty liver [26]; analysis of mechanistic aspects of this phenomenon has revealed involvement of the pro-inflammatory cytokine interleukin-6 in the upregulation of SLC13A5 in hepatocytes in association with fatty liver.

## 6. Biochemical and Metabolic Phenotype of *Slc13a5*-Knockout Mouse

In vitro studies have demonstrated that NaCT promotes the conversion of extracellular citrate into lipids in liver cell lines [20]. Studies with *Slc13a5*-knockout mice corroborate a similar function in vivo [27]. The knockout mice are viable with no overt phenotype. However, biochemically and metabolically, these mice exhibit a phenotype akin to animals undergoing caloric restriction. First of all, the hepatic levels of ATP are decreased, indicating suppressed mitochondrial function. This decrease in cellular energy is accompanied with increased activity of hepatic AMPK (AMP-activated protein kinase), a cellular marker for energy deficiency. In addition, fatty acid synthesis goes down as evident from the decreased activity of acetyl CoA carboxylase-2, the enzyme that converts acetyl CoA into malonyl CoA as the first step in fatty acid biosynthesis. The mice also exhibit increased activity for fatty acid oxidation and energy expenditure. The increase in fatty acid oxidation in the knockout mice is at least in part related to a decrease in the cellular levels of malonyl CoA. Oxidation of long-chain fatty acids within the mitochondria depends on the import of these molecules from the cytoplasm into the mitochondrial matrix across the inner mitochondrial membrane, which requires the enzyme carntine-palmitoyl transferase-1 (CPT-1); this enzyme is inhibited by malonyl CoA. As the cellular levels of malonyl CoA are decreased in the hepatocytes of the knockout mice, the import of long-chain fatty acids into the mitochondrial matrix is facilitated, thus resulting in increased activity of fatty acid oxidation. The ability of these hepatocytes to metabolize glucose via glycolysis is also most likely enhanced, even though this has not yet been documented experimentally. The rationale for this speculation is that citrate is a potent inhibitor of phosphofructokinase-1, an important rate-limiting enzyme in glycolysis. The deletion of Slc13a5 is expected to reduce the cytoplasmic levels of citrate, thus relieving the inhibition of this enzyme. Collectively, these biochemical features are expected to protect against metabolic syndrome. This is indeed the case; the knockout mice are resistant to high-fat-induced adiposity, body weight gain, fatty liver, and insulin resistance. A similar metabolic phenotype also occurs in rats when Slc13a5 is silenced with antisense oligonucleotides [28].

These findings with mice and rats indicate that pharmacological inhibition of NaCT might have therapeutic potential in the prevention of diet-induced obesity, hyperlipidemia, insulin resistance, and diabetes. With this rationale, several laboratories are focusing on identifying selective high-affinity inhibitors of NaCT [29,30]; these studies have identified small molecules with ability to inhibit NaCT at submicromolar concentrations.

## 7. Functional Loss of SLC13A5 as a Cause of Epilepsy in Humans

NaCT was first cloned from rat brains [14]; subsequent studies showed that the transporter is also expressed in mouse brains and human brains [15,16]. The expression is mostly restricted to neurons [18]. In addition to citrate present in circulation, astrocytes release citrate into the extracellular medium [31,32]; therefore, it is logical to speculate that the physiological function of NaCT in neurons is to take up citrate for their metabolic use. However, *Slc13a5*-knockout mice do not show any evidence of neuronal dysfunction [26], thereby suggesting that the transporter does not play any obligatory role in neurons. Recent studies have shown that this is certainly not the case in humans. Loss-of-function mutations in SLC13A5 are associated with early onset epilepsy in children; epileptic symptoms appear within the first few weeks after birth and seizures persist even as the affected children grow [33,34,35]. SLC13A5-associated epilepsy is unique in a number of ways. First, it is accompanied with developmental delay, slow progression of motor function, and significant impairment in language and speech development. Second, it does not respond to ketogenic diet or low calorie intake; the patients, however, seem to show some improvement in response to drugs that affect GABA signaling [35]. Third, defects in teeth development, identified as microdontia, are seen in some of these children [34]. Fourth, this is an autosomal recessive disease with no noticeable phenotype in heterozygote carriers.

## 8. Molecular Mechanisms Underlying SLC13A5-Associated Epilepsy

Virtually nothing is known on the molecular basis for the development of epilepsy in children with loss-of-function mutations in SLC13A5 except that the association between SLC13A5 mutations and epilepsy certainly indicates an obligatory function for the transporter in the brain. There are several potential mechanisms by which a complete lack of the transport function of NaCT would cause neuronal dysfunction and epilepsy. The first mechanism that comes to mind is the relevance to NaCT function to neuronal energetics. Citrate is an important energy-rich molecule (Table 1); even though glucose is the primary source of biological energy in the brain, other metabolites might contribute to energy production in neurons to a significant extent. It is well known that ketone bodies such as β-hydroxybutyrate serve as an important alternative energy source to the brain when glucose is not available or cannot be metabolized as occurs in prolonged starvation or uncontrolled diabetes [36,37]. Citrate might serve as a significant source of energy in neurons, suggesting that loss of function of NaCT might lead to energy deficit, thereby precipitating delayed brain development and epilepsy. This would be akin to epilepsy resulting from loss-of-function mutations in the facilitative glucose transporter GLUT1 (SLC2A1) [38]. A complete loss of GLUT1 function is incompatible with life, but heterozygous loss of GLUT1 function causes epileptic seizures. This illustrates the importance of glucose as the energy source to the brain. GLUT1 is expressed in the blood–brain barrier and is the sole mechanism for the transfer of glucose from circulation into brain parenchymal tissue. However, the theory of energy deficiency as the cause of epilepsy in children with loss-of-function mutations in NaCT has two significant, almost insurmountable, flaws. First, in many cases, the SLC13A5-associated epilepsy does not respond to ketogenic diets that provide alternative energy sources to the brain. This treatment works successfully in children suffering from GLUT1 transport defects. If SLC13A5-associated epilepsy arises solely due to insufficient amount of energy in neurons, why doesn’t it respond to the administration of alternative energy substrates? Second, citrate would produce energy only when present within the mitochondrial matrix; even though the citrate transporter in the inner mitochondrial membrane normally functions in the transfer of citrate from the matrix into the cytoplasm, it could mediate the entry of cytoplasmic citrate into the matrix for subsequent metabolism in the tricarboxylic acid cycle if the concentrations of citrate in the cytoplasm are sufficiently high. It is not known whether this happens under any physiological condition. Furthermore, loss-of-function mutations in the mitochondrial citrate transporter (SLC25A1) also cause epilepsy [39,40]. As citrate generates energy solely within the mitochondrial matrix, why would defects in the mitochondrial citrate transporter cause epilepsy? Such defects would lead to decreased release of citrate from the matrix, thus maximizing the use of citrate for energy production. These arguments question the validity of the idea that defects in SLC25A1 cause epilepsy because of decreased energy production. The correct explanation must therefore lie somewhere else in citrate metabolism in neuronal cells.

## 9. The Cytoplasmic Citrate Deficit Hypothesis: Mitochondrial Citrate versus Cytoplasmic Citrate in Relation to Epilepsy

**Figure 2:**
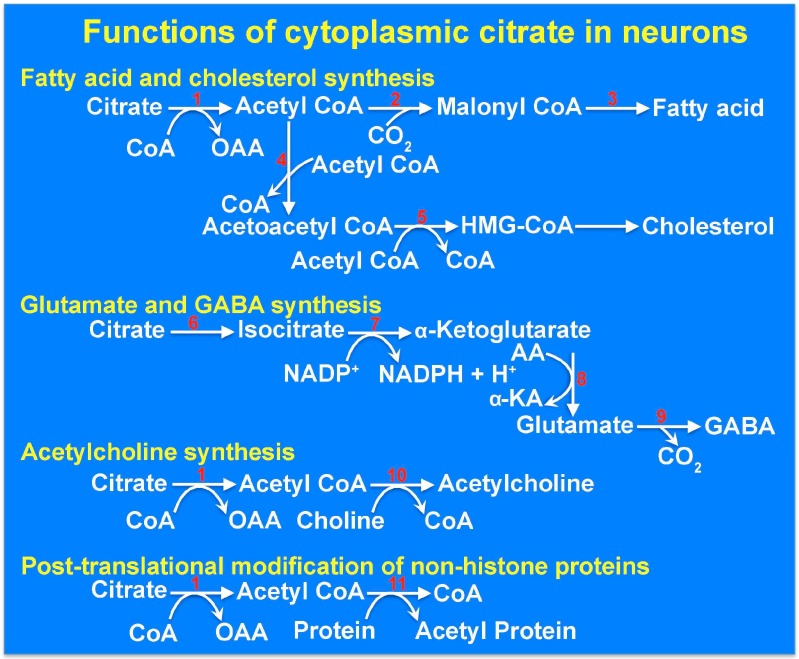
Functions of citrate in the cytoplasm of neurons. The enzymes listed are: 1: ATP:citrate lyase; 2: Acetyl CoA carboxylase; 3: Fatty acid synthase complex; 4: Thiolase; 5: HMG CoA synthase; 6: Aconitase; 7: Isocitrate dehydrogenase; 8: Transaminase; 9: Glutamate decarboxylase; 10: Choline acetyl transferase; 11: Protein (histone) acetyl transferases. CoA, coenzyme A; OAA, oxaloacetate; AA, amino acid; α-KA, α-Ketoacid; GABA, γ-aminobutyrate; HMG CoA, 3-hydroxy-3-methyl glutaryl CoA.

Until the discovery of the plasma membrane transporter NaCT, it was assumed that the cytoplasmic citrate that participates in fatty acid and cholesterol biosynthesis solely originates from the mitochondrial matrix. Now, we know that citrate in the cytoplasm has two sources: citrate generated inside the mitochondria that is transported out into the cytoplasm via the mitochondrial citrate transporter SLC25A1, and citrate present in the circulation and extracellular fluid that is transported into the cytoplasm via the plasma membrane citrate transporter SLC13A5 (Figure 1). Even though a recent report on model-based assessment of citrate flux from plasma into liver cells has concluded that only less than 10% of hepatic citrate comes from plasma [41], other studies have shown that extracellular citrate contributes significantly to fatty acid biosynthesis in liver cells [20,26]. In addition to the citrate present in plasma and extracellular medium in the brain, cerebrospinal fluid also contains high levels of citrate (~500 μM) [42], but there is no information available in the literature as to expression of the plasma membrane citrate transporter SLC13A5 in epithelial cells of choroid plexus. Loss of function of either SLC13A5 or SLC25A1 causes epilepsy even though the consequences of their loss of function on mitochondrial citrate levels are opposite. Whereas SLC25A1 defects increase mitochondrial citrate levels, SLC13A5 defects are not expected to elicit a similar effect. However, defects in both transporters would decrease cytoplasmic citrate levels. It is therefore tempting to speculate that it is the deficiency of citrate in the cytoplasm of neuronal cells that underlies the pathogenesis of this particular form of epilepsy. One of the well-documented functions of citrate in the cytoplasm is in the regulation of glycolysis where it allosterically inhibits the rate-limiting enzyme phosphofructokinase-1 and suppresses glycolysis. This particular function is not likely to be relevant to the pathogenesis of epilepsy for two reasons. First, decreased levels of citrate in the cytoplasm would be expected to increase glycolysis and hence energy production, an unlikely cause of epilepsy. Second, metabolic pathways are compartmentalized between astrocytes and neurons in the brain; when neurons are activated, astrocytes take up glucose and metabolize it to lactate, which is then released into the extracellular fluid for subsequent uptake by neurons for use in energy production [43,44]. As glycolysis occurs primarily in astrocytes rather than in neurons whereas the citrate transporter SLC13A5 is expressed only in neurons, it minimizes the potential relevance of the role of citrate as a regulator of glycolysis in neurons. The other functions of citrate in the cytoplasm include its role as a sole source of carbon in the biosynthesis of fatty acids and cholesterol in the generation of the neurotransmitters glutamate and γ-aminobutryate and in acetylation of histones and non-histone proteins [45] (Figure 2). A decrease in the cytoplasmic levels of citrate, which is expected to happen as a consequence of SLC13A5 defects, would compromise all these functions. Decreased synthesis of fatty acids and cholesterol might interfere with neuronal myelination. Glutamate can arise from citrate in the cytoplasm by the sequential actions of aconitase, isocitrate dehydrogenase, and transaminases; all of these enzyme activities are present not only in the mitochondrial matrix but also in the cytoplasm. Glutamic acid decarboxylase would then convert glutamate into γ-aminobutyrate (GABA) in GABAergic neurons. Dysfunction of this GABA-generating enzyme is associated with epilepsy in mice [46]. It is therefore possible that loss-of-function mutations in SLC13A5 decrease cytoplasmic levels of citrate and consequently decrease the synthesis of the inhibitory neurotransmitter GABA. Likewise, alterations in acetylation status of metabolic enzymes in the cytoplasm resulting from decreased citrate and acetyl CoA might also contribute to the pathogenesis of epilepsy. For example, ATP:citrate lyase, which is critical for the synthesis of fatty acids from cytoplasmic citrate is activated by acetylation [47]; this indicates that reduced levels of citrate in the cytoplasm not only decrease the availability of the carbon source but could also decrease the conversion of citrate into acetyl CoA in the metabolic pathway involved in fatty acid biosynthesis by preventing acetylation of this key enzyme. Citrate in the cytoplasm might also influence the synthesis of another neurotransmitter, acetylcholine. Any or all of these mechanisms might potentially underlie the pathogenesis of epilepsy in patients with SLC13A5 deficiency. Further studies are needed to tease out individually the relevance of these varied functions of cytoplasmic citrate to epilepsy. Only then would we be able to come up with appropriate therapeutic strategies to treat epilepsy in children affected with the loss-of-function mutations in SLC13A5. If a decrease in the cytoplasmic levels of citrate is indeed the cause of epilepsy in these children, the focus for treatment should be on restoring the citrate levels in the cytoplasm of the neurons by some means bypassing the defective plasma membrane citrate transporter.

## 10. The Interneuron Energy Hypothesis: A Mechanism Linking Low Cytoplasmic Citrate to Seizure Susceptibility

There is growing recognition that mutations in genes associated with energy metabolism, such as mitochondrial proteins associated with the TCA cycle, are associated with epilepsy [48,49]. Since an imbalance between excitatory glutamatergic and inhibitory GABAergic neurons must exist to induce epileptogenesis, it is not immediately obvious how an energy deficit affecting common metabolic neuronal machinery would induce such an imbalance. However, of neurons in the brain, inhibitory neurons have a higher energy metabolism than principal neurons [50]. During periods of intense neural activity, for example, when inhibitory neurons are driven by glutamatergic inputs during gamma oscillations, considerable cellular energy expenditure is needed to clear ionic gradients disrupted during synaptic transmission. Because of their energy usage, inhibitory circuits could become vulnerable under conditions of reduced energy availability, which has been coined the Interneuron Energy Hypothesis [50]. If an energy deficit is generated in inhibitory neurons through reduced cytoplasmic citrate availability, the ability of inhibitory neurons to restore ionic gradients could be impaired, which could lead to excitation–inhibition imbalance and seizure susceptibility.

## 11. The Zinc Chelation Hypothesis: A Mechanism Linking High Extracellular Citrate to Seizure Susceptibility

**Figure 3:**
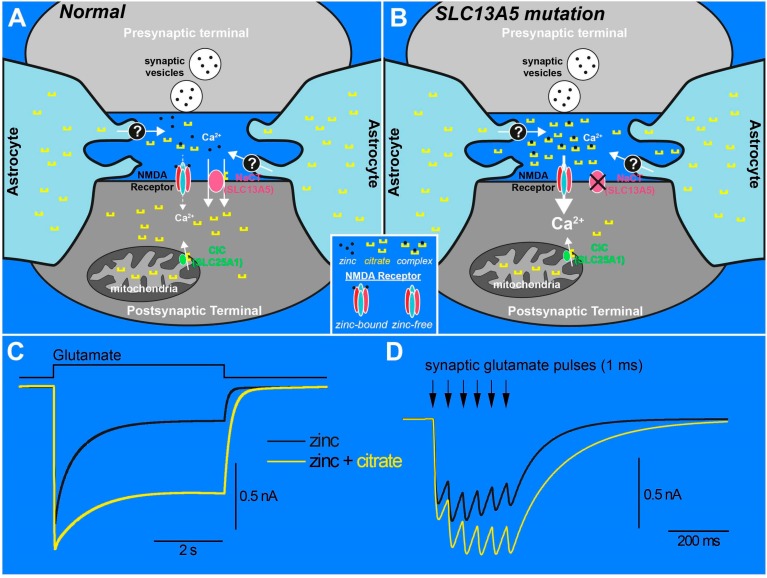
The possible role of zinc and NMDA receptors in loss-of-function SLC13A5 mutations. (**A**) normal condition. Astrocytes release citrate (yellow U-shaped symbols) into the extracellular space via an unknown mechanism (denoted by a question mark). The extracellular zinc binding site on the NR2A subunit (red) of the NMDA receptor is occupied by zinc ions (black filled circles), acting as a check on NMDA receptor-induced calcium influx. The plasma membrane citrate transporter NaCT (SLC13A5, pink) limits the accumulation of extracellular citrate by transporting citrate into the cytoplasm. Citrate extrusion from the mitochondria via the mitochondrial citrate carrier (CIC or SLC25A1, green) is also a source of cytosolic citrate; (**B**) When SLC13A5 function is lost, intracellular citrate levels are likely reduced while extracellular citrate levels rise to pathologically high levels. Citrate chelation of free zinc reduces the availability of zinc for the NMDA receptor, relieving the negative allosteric effect of zinc on NMDA receptor function, thereby increasing calcium flux through the NMDA receptor (note larger arrow indicating enhanced flux of calcium through the NMDA receptor). Schematics in A and B were motivated from [30,61]; (**C**) kinetic model simulations [55,62] of NMDA receptor currents generated by a 5 s pulse of glutamate under normal conditions (i.e., with free zinc) and under conditions in which extracellular citrate has chelated free zinc (i.e., without free zinc). Citrate chelation induces a larger and more sustained NMDA receptor current; (**D**) response of the kinetic model to six synaptic-like 1-ms pulses of glutamate, simulating a burst of gluatmatergic synaptic activity. Note that NMDA receptor-mediated currents summate and deactivate more slowly under conditions of elevated extracellular citrate levels than under the normal conditions. These simulations demonstrate that increased extracellular citrate levels have the potential to enhance NMDA receptor-mediated synaptic transmission, which is likely to yield pro-convulsant effects.

Citrate is released from astrocytes to the extracellular space and is normally transported into the cytoplasm of neurons through the action of SLC13A5 [18,31,32]. Although a loss-of-function mutation in SLC13A5 is likely to have adverse consequences on cytoplasmic citrate levels in neurons, it is also likely that extracellular citrate levels will rise. Interestingly, citric acid injection into the cerebrospinal fluid has been shown to induce seizures in rodents, which is inhibited by co-administration of Ca^2+^ [51]. Citrate is a potent chelator of divalent cations, including Mg^2+^ and Ca^2+^, and particularly Zn^2+^ [52,53]. The NR2A subunit of the NMDA receptor possesses a high-affinity allosteric binding site for zinc [54,55,56]. Occupation of this high affinity site by Zn^2+^ has an inhibitory effect on NMDA receptor function [54,55,56]. In a direct demonstration of the chelating effects of zinc on NMDA receptor function, Westergaard and colleagues showed that co-application of citrate with free zinc enhances NMDA receptor-mediated currents [57]. In an elegant study, Vergnano and colleagues demonstrated that NMDA receptor-mediated synaptic currents are potentiated under conditions in which zinc is deficient from synaptic vesicles, the high-affinity zinc-binding site is eliminated from NR2A receptors, or zinc is chelated [58]. The effects of zinc chelation are most prominent at glutamatergic synapses that synaptically release zinc and possess NR2A-containing synapses. In addition, since zinc is contained in synaptic vesicles and is released synaptically [58,59], prolonged synaptic stimulation tends to enhance the effects of zinc, and, therefore, effects of zinc chelators, at synaptic NMDA receptors. Therefore, the idea that zinc chelation could potentiate NMDA receptor function at central glutamatergic synapses is a plausible mechanism that could explain how elevated citrate levels may lead to enhanced NMDA receptor-mediated synaptic transmission (Figure 3).

Is reduced zinc occupancy at NMDA receptors associated with an epilepsy phenotype? Recently, studies have shown that human mutations that alter zinc affinity on NR2A-containing NMDA receptors are associated with childhood epilepsies and cognitive deficits [60]. Decreased Zn^2+^ serum levels have also been linked to febrile seizures in both humans and animal models [61]. In the context that enhanced extracellular citrate is convulsive [51], it seems likely that reduced zinc levels through citrate chelation are a mechanism that could at least partially contribute to excitation–inhibition imbalance and seizure susceptibility.

## 12. Conclusions

SLC13A5 is a transporter in the plasma membrane that mediates Na^+^-coupled active uptake of citrate into cells. It is expressed in hepatocytes, neurons, and spermatozoa. Recent studies have shown that loss-of-function mutations in the gene coding for this transporter are associated with neonatal epilepsy in humans. This is a single-gene disease with epilepsy resulting solely from the inactivity of SLC13A5. The molecular mechanisms underlying the pathogenesis of neuronal dysfunction and epilepsy in this disease remain largely unknown, thus preventing a rational approach for the design and development of effective treatment strategies. There are several potential mechanisms, but more research is needed to delineate the possible involvement of each of these pathways.

## Figures and Tables

**Table 1:** Concentration and energy content of tricarboxylic acid cycle intermediates in plasma.

Intermediates	Concentration
Tricarboxylates	
Citrate (10 ATP/mole)	~160 μM
Isocitrate (10 ATP/mole)	<10 μM
Dicarboxylates	
α-Ketoglutarate (7.5 ATP/mole)	~10 μM
Succinate (4 ATP/mole)	~40 μM
Fumarate (2.5 ATP/mole)	<10 μM
Malate (2.5 ATP/mole)	~35 μM
Oxaloacetate	<10 μM
Monocarboxylates	
Lactate (15 ATP/mole)	~1 mM
Pyruvate (12.5 ATP/mole)	~70 μM

## References

[1] Hodgkinson A. (1963). The relation between citric acid and calcium metabolism with particular reference to primary hyper-parathyroidism and idiopathic hypercalcemia. Clin. Sci..

[2] Fraenkl S.A., Muser J., Groell R., Reinhard G., Orgul S., Flammer J., Goldblum D. (2011). Plasma citrate levels as a potential biomarker for glaucoma. J. Ocul. Pharmacol. Ther..

[3] Halestrap A.P. (2013). The SLC16 gene family—Structure, role and regulation in health and disease. Mol. Asp. Med..

[4] Wright E.M. (2013). Glucose transport families SLC5 and SLC50. Mol. Asp. Med..

[5] Ganapathy V., Thangaraju M., Gopal E., Martin P.M., Itagaki S., Miyauchi S., Prasad P.D. (2008). Sodium-coupled monocarboxylate transporters in normal tissues and in cancer. AAPS J..

[6] Bergeron M.J., Clemencon B., Hediger M.A., Markovich D. (2013). SLC13 family of Na^+^-coupled di- and tri-carboxylate/sulfate transporters. Mol. Asp. Med..

[7] Rogina B., Reenan R.A., Nilsen S.P., Helfand S.L. (2000). Extended life-span conferred by cotransporter gene mutations in Drosophila. Science.

[8] Sohal R.S., Forster M.J. (2014). Caloric restriction and the aging process: A critique. Free Radic. Biol. Med..

[9] Ruetenik A., Barrientos A. (2015). Dietary restriction, mitochondrial function and aging: From yeast to humans. Biochim. Biophys. Acta.

[10] Willmes D.M., Birkenfeld A.L. (2013). The role of INDY in metabolic regulation. Comput. Struct. Biotechnol..

[11] Rogers R.P., Rogina B. (2015). The role of INDY in metabolism, health and longevity. Front. Genet..

[12] Inoue K., Fei Y.J., Huang W., Zhuang L., Chen Z., Ganapathy V. (2002). Functional identity of Drosophila melanogaster Indy as a cation-independent, electroneutral transporter for tricarboxylic acid-cycle intermediates. Biochem. J..

[13] Knauf F., Rogina B., Jiang Z., Aronson P.S., Helfand S.L. (2002). Functional characterization and immunolocalization of the transporter encoded by the life-extending gene Indy. Proc. Natl. Acad. Sci. USA.

[14] Inoue K., Zhuang L., Maddox D.M., Smith S.B., Ganapathy V. (2002). Structure, function, and expression pattern of novel sodium-coupled citrate transporter (NaCT) cloned from mammalian brain. J. Biol. Chem..

[15] Inoue K., Zhuang L., Ganapathy V. (2002). Human Na^+^-coupled citrate transporter: Primary structure, genomic organization, and transport function. Biochem. Biophys. Res. Commun..

[16] Inoue K., Fei Y.J., Zhuang L., Gopal E., Miyauchi S., Ganapathy V. (2004). Functional features and genomic organization of mouse NaCT, a sodium-coupled transporter for tricarboxylic acid cycle intermediates. Biochem. J..

[17] Gopal E., Babu E., Ramachandran S., Bhutia Y.D., Prasad P.D., Ganapathy V. (2015). Species-specific influence of lithium on the activity of SLC13A5 (NaCT): Lithium-induced activation is specific for the transporter in primates. J. Pharmacol. Exp. Ther..

[18] Yodoya E., Wada M., Shimada A., Katsukawa H., Okada N., Yamamoto A., Ganapathy V., Fujita T. (2006). Functional and molecular identification of sodium-coupled dicarboxylate transporters in rat primary cultured cerebrocortical astrocytes and neurons. J. Neurochem..

[19] Gopal E., Miyauchi S., Martin P.M., Ananth S., Srinivas S.R., Smith S.B., Prasad P.D., Ganapathy V. (2007). Expression and functional features of NaCT, a sodium-coupled citrate transporter, in human and rat livers and cell lines. Am. J. Physiol. Gastrointest. Liver Physiol..

[20] Inoue K., Zhuang L., Maddox D.M., Smith S.B., Ganapathy V. (2003). Human sodium-coupled citrate transporter, the orthologue of Drosophila Indy, as a novel target for lithium action. Biochem. J..

[21] Chen Y., Silverstone T. (1990). Lithium and weight gain. Int. Clin. Psycopharmacol..

[22] Baptista T., Teneud L., Contreras Q., Alastre T., Burguera J.L., de Burguera M., de Baptista E., Weiss S., Hernandez L. (1995). Lithium and body weight gain. Pharmacopsychiatry.

[23] Li L., Li H., Gaszei B., Yang H., Sueyoshi T., Li Q., Shu Y., Zhang J., Hu B., Heyward S. (2015). SLC13A5 is a novel transcriptional target of the pregnane X receptor and sensitizes drug-induced steatosis in human liver. Mol. Pharmacol..

[24] Neuschafer-Rube F., Schraplau A., Schewe B., Lieske S., Krutzfeldt J.M., Ringel S., Henkel J., Birkenfeld A.L., Puschel G.P. (2015). Arylhydrocarbon receptor-dependent mIndy (Slc13a5) induction as possible contributor to benzo[*a*]pyrene-induced lipid accumulation in hepatocytes. Toxicology.

[25] Neuschafer-Rube F., Lieske S., Kuna M., Henkel J., Perry R.J., Erion D.M., Pesta D., Willmes D.M., Brachs S., von Loeffelholz C. (2014). The mammalian INDY homolog is induced by CREB in a rat model of type 2 diabetes. Diabetes.

[26] Von Loeffelholz C., Lieske S., Neuschafer-Rube F., Willmes D.M., Raschzok N., Sauer I.M., Konig J., Fromm M., Horn P., Chatzigeorgiou A. (2017). The human longevity gene homolog INDY and interleukin-6 interact in hepatic lipid metabolism. Hepatology.

[27] Birkenfeld A.L., Lee H.Y., Guebre-Egziabher F., Alves T.C., Jurczak M.J., Jornayvaz F.R., Zhang D., Hsiao J.J., Martin-Montalvo A., Fischer-Rosinsky A. (2011). Deletion of the mammalian INDY homology mimics aspects of dietary restriction and protects against adiposity and insulin resistance in mice. Cell Metab..

[28] Pesta D.H., Perry R.J., Guebre-Egziabher F., Zhang D., Jurczak M., Fischer-Rosinsky A., Daniels M.A., Willmes D.M., Bhanot S., Bornstein S.R. (2015). Prevention of diet-induced hepatic steatosis and hepatic insulin resistance by second generation antisense oligonucleotides targeted to the longevity gene mIndy (Slc13a5). Aging.

[29] Huard K., Brown J., Jones J.C., Cabral S., Futatsugi K., Gorgoglione M., Lanba A., Vera N.B., Zhu Y., Yan Q. (2015). Discovery and characterization of novel inhibitors of the sodium-coupled citrate transporter (NaCT or SLC13A5). Sci. Rep..

[30] Huard K., Gosset J.R., Montgomery J.I., Gilbert A., Hayward M.M., Magee T.V., Cabral S., Uccello D.P., Bahnck K., Purkal J. (2016). Optimization of a dicarboxylic series for in vivo inhibition of citrate transport by the solute carrier 13 (SLC13) family. J. Med. Chem..

[31] Sonnewald U., Westergaard N., Krane J., Unsgard G., Petersen S.B., Schousboe A. (1991). First direct demonstration of preferential release of citrate from astrocytes using [^13C^]NMR spectroscopy of cultured neurons and astrocytes. Neurosci. Lett..

[32] Mycielska M.E., Milenkovic V.M., Wetzel C.H., Rummele P., Geissler E.K. (2015). Extracellular citrate in health and disease. Curr. Mol. Med..

[33] Thevenon J., Milh M., Feillet F., St-Onge J., Duffourd Y., Juge C., Roubertie A., Heron D., Mignot C., Raffo E. (2014). Mutations in SLC13A5 cause autosomal-recessive epileptic encephalopathy with seizure onset in the first days of life. Am. J. Hum. Genet..

[34] Hardies K., de Kovel C.G., Weckhuysen S., Asselbergh B., Geuens T., Deconinck T., Azmi A., May P., Brilstra E., Becker F. (2015). Recessive mutations in SLC13A5 result in a loss of citrate transport and cause neonatal epilepsy, developmental delay and teeth hypoplasia. Brain.

[35] Klotz J., Porter B.E., Colas C., Schlessinger A., Pajor A.M. (2016). Mutations in the Na^+^/citrate cotransporter NaCT (SLC13A5) in pediatric patients with epilepsy and developmental delay. Mol. Med..

[36] McNally M.A., Hartman A.L. (2012). Ketone bodies in epilepsy. J. Neurochem..

[37] Lauritzen F., Eid T., Bergersen L.H. (2015). Monocarboxylate transporters in temporal lobe epilepsy: Roles of lactate and ketogenic diet. Brain Struct. Funct..

[38] Wolking S., Becker F., Bast T., Wiemer-Kruel A., Mayer T., Lerche H., Weber Y.G. (2014). Focal epilepsy in glucose transporter type 1 (Glut1) defects: Case reports and a review of literature. J. Neurol..

[39] Muhlhausen C., Salomons G.S., Lukacs Z., Struys E.A., van der Knaap M.S., Ullrich K., Santer R. (2014). Combined D_2_-/L_2_-hydroxyglutaric aciduria (SLC25A1 deficiency): Clinical course and effects of citrate treatment. J. Inherit. Metab. Dis..

[40] Smith A., MaBride S., Marcadier J.L., Michaud J., Al-Dirbashi O.Y., Schwartzentruber J., Beaulieu C.L., Katz S.L., Majewski J., FORGE Canada Consortium (2016). Severe neonatal presentation of mitochondrial citrate carrier (SLC25A1) deficiency. JIMD Rep..

[41] Li Z., Erion D.M., Maurer T.S. (2016). Model-based assessment of plasma citrate flux into the liver: Implications for NaCT as a therapeutic target. CPT Pharmacomet. Syst. Pharmacol..

[42] Ramautar R., Somsen G.W., de Jong G.J. (2007). Direct sample injection for capillary electrophoretic determination of organic acids in cerebrospinal fluid. Anal. Bioanal. Chem..

[43] Pellerin L. (2008). Brain energetics (thought needs food). Curr. Opin. Clin. Nutr. Metab. Care.

[44] Dienel G.A., Cruz N.F. (2016). Aerobic glycolysis during brain activation: Adrenergic regulation and influence of norepinephrine on astrocytic metabolism. J. Neurochem..

[45] Iacobazzi V., Infantino V. (2014). Citrate—New functions for an old metabolite. Biol. Chem..

[46] Kash S.F., Johnson R.S., Tecott L.H., Noebels J.L., Mayfield R.D., Hanahan D., Baekkeskov S. (1997). Epilepsy in mice deficient in the 65-kDa isoform of glutamic acid decarboxylase. Proc. Natl. Acad. Sci. USA.

[47] Lin R., Tao R., Gao X., Li T., Zhou X., Guan K.L., Xiong Y., Lei Q.Y. (2013). Acetylation stabilizes ATP-citrate lyase to promote lipid biosynthesis and tumor growth. Mol. Cell.

[48] Kovac S., Abramov A.Y., Walker M.C. (2013). Energy depletion in seizures: Anaplerosis as a strategy for future therapies. Neuropharmacology.

[49] Zsurka G., Kunz W.S. (2015). Mitochondrial dysfunction and seizures: The neuronal energy crisis. Lancet Neurol..

[50] Kann O. (2016). The interneuron energy hypothesis: Implications for brain disease. Neurobiol. Dis..

[51] Hornfeldt C.S., Larson A.A. (1990). Seizures induced by fluoroacetic acid and fluorocitric acid may involve chelation of divalent cations in the spinal cord. Eur. J. Pharmacol..

[52] Field T., Coburn J., McCourt J., McBryde W.A.E. (1975). Composition and stability of some metal citrate and diglycolate complexes in aqueous solution. Anal. Chim. Acta.

[53] Glusker J. (1980). Citrate conformation and chelation: Enzymic implications. Acc. Chem. Res..

[54] Paoletti P., Ascher P., Neyton J. (1997). High-affinity zinc inhibition of NMDA NR1-NR2A receptors. J. Neurosci..

[55] Amico-Ruvio S., Murthy S., Smith T., Popescu G. (2011). Zinc effects on NMDA receptor gating kinetics. Biophys. J..

[56] Romero-Hernandez A., Simorowski N., Karakas E., Furukawa H. (2016). Molecular basis for subtype specificity and high-affinity zinc inhibition in the GluN1-GluN2A NMDA receptor amino-terminal domain. Neuron.

[57] Westergaard N., Banke T., Wahl P., Sonnewald U., Schousboe A. (1995). Citrate modulates the regulation by Zn^2+^ of *N*-methyl-d-aspartate receptor-mediated channel current and neurotransmitter release. Proc. Natl. Acad. Sci. USA.

[58] Vergnano A.M., Rebola N., Savtchenko L.P., Pinheiro P.S., Casado M., Kieffer B.L., Rusakov D.A., Mulle C., Paoletti P. (2014). Zinc dynamics and action at excitatory synapses. Neuron.

[59] Tóth K. (2011). Zinc in neurotransmission. Nutrition.

[60] Serraz B., Grand T., Paoletti P. (2016). Altered zinc sensitivity of NMDA receptors harboring clinically-relevant mutations. Neuropharmacology.

[61] Reid C., Hildebrand M., Mullen S., Hildebrand J., Berkovic S., Petrou S. (2017). Synaptic Zn^2+^ and febrile seizure susceptibility. Br. J. Pharmacol..

[62] Goldschen-Ohm M., Haroldson A., Jones M., Pearce R. (2014). A nonequilibrium binary elements-based kinetic model for benzodiazepine regulation of GABAA receptors. J. Gen. Physiol..

